# Genotypic and phenotypic analysis of diarrheagenic *Escherichia coli* strains isolated from Brazilian children living in low socioeconomic level communities

**DOI:** 10.1186/1471-2334-13-418

**Published:** 2013-09-08

**Authors:** Diego M Lozer, Tamara B Souza, Mariane V Monfardini, Fernando Vicentini, Sônia S Kitagawa, Isabel C A Scaletsky, Liliana C Spano

**Affiliations:** 1 Departamento de Patologia, Universidade Federal do Espirito Santo, Espirito Santo, Brazils; 2 Departamento de Microbiologia, Imunologia e Parasitologia, Universidade Federal de São Paulo, Rua Botucatu, 862, 3 andar, 04023-062, São Paulo, Brazil; 3 Departamento de Ciências da Saúde, Universidade Federal do Espírito Santo, Espírito Santo, Brazil

## Abstract

**Background:**

Childhood diarrheal diseases remain highly endemic in developing areas of Brazil. The importance of *Escherichia coli* among children with diarrhea in these areas was unknown. This study determined the prevalence of different *E. coli* categories in symptomatic and asymptomatic children from low socioeconomic level rural communities in southeastern Brazil.

**Methods:**

A total of 560 stool samples were collected from 141 children with diarrhea (< 10 years) and 419 apparently healthy controls who resided in 23 communities. *E. coli* isolates (n = 1943) were subjected to two multiplex PCRs developed for the detection of enteropathogenic *E. coli* (EPEC), enteroaggregative *E. coli* (EAEC), diffusely adherent *E. coli* (DAEC), enterotoxigenic *E. coli* (ETEC), enteroinvasive *E. coli* (EIEC), and Shiga toxin-producing *E. coli* (STEC). Strains were also examined for the presence of EPEC, EAEC, and DAEC by assays of adhesion to HEp-2 cells and by hybridization with specific DNA probes.

**Results:**

Diarrheagenic *E. coli* strains were isolated from 253 (45.2%) children, and were associated with diarrhea in children aged < 5 years (p < 0.001). EAEC (20.9%), DAEC (11.6%), EPEC (9.3%) were the most frequent pathotypes, followed by ETEC (2.7%), EIEC (0.5%), and STEC (0.2%). Depending of the assay, EPEC, EAEC, and DAEC (collectively termed enteroadherent *E. coli)* strains were isolated in 45% to 56% of diarrhea cases, a significantly higher incidence than in controls (*P* < 0.05). Individually, only DAEC showed significant association with diarrhea (p < 0.05), particularly in children aged 2–5 years.

**Conclusion:**

This study indicates that enteroadherent *E. coli* is an important cause of diarrhea in children living in low socioeconomic level communities in southeastern Brazil. Our results reveal that the PCR1 assay is an excellent tool for the identification of EAEC and DAEC.

## Background

Diarrheal disease remains a major public health problem in developing countries, and is responsible for high morbidity and mortality among children under 5 years [1,2]. In Latin America, diarrheal diseases are responsible for ~10% of childhood deaths [3]. *Escherichia coli* strains are among the most important bacterial causes of childhood diarrhea. Diarrheagenic *E. coli* (DEC) strains can be divided into six main categories on the basis of distinct epidemiological and clinical features, and specific virulence determinants [4]: enteropathogenic *E. coli* (EPEC), enterotoxigenic *E. coli* (ETEC), enteroinvasive *E. coli* (EIEC), enterohemorrhagic *E. coli* (EHEC) or Shiga-toxin producing *E. coli* (STEC), enteroaggregative *E. coli* (EAEC), and diffusely adherent *E. coli* (DAEC). EPEC is classified into typical and atypical strains based on the presence of the EPEC adherence factor (EAF) plasmid [4]. Although DEC strains are of public health relevance, they are not routinely sought as enteric pathogens in clinical laboratories worldwide; thus the epidemiology of DEC infections remains obscure in many parts of world, particularly in endemic areas. Some assays for the detection of DEC are available, such as biochemical reactions, serotyping, phenotypic assays based on virulence characteristics, and molecular methods [4]. Among these, PCR is a commonly used method that gives rapid, reliable results with a high sensitivity and a high specificity. In order to simply diagnosis, we set up a two-reaction multiplex PCR assay for the detection of EPEC, EAEC, DAEC, ETEC, EIEC, and STEC, but so far has not been used in field studies [5].

In recent studies conducted in different urban centers of Brazil, aEPEC strains were found to be dominant and associated with diarrhea [6-9]. These studies also identified EAEC and DAEC in children with diarrhea. We conducted a prospective active diarrhea surveillance study to determine the importance of EPEC, EAEC and DAEC in rural communities with low socioeconomic level in southeastern Brazil using phenotypic and genotypic diagnostic tests.

## Methods

### Ethical clearance, study site and population

The study was approved by the Ethical Committee of Research (reference number 129/06) in the Health Science of Center at the Universidade Federal do Espírito Santo, Brazil. A consent form was read and signed by the parents or guardians of each child. The study was conducted in “quilombola” communities with low socioeconomic status located 256 km north of the city of Espirito Santo, Brazil. The “quilombola” communities are composed of descendents of slaves who escaped from slave plantations that existed in Brazil before abolition in 1880. These communities are predominately agricultural, and the population lives in slum conditions, with no access to potable water or sewage facilities. The households were visited once a week by nurses and trained community health workers. At each visit, the mother was asked a standard series of questions on her child´s stool frequency, consistency, and physical nature. If a child had diarrhea, his or her hydration status was assessed. Generally fluid and nutritional management was recommended. Diarrhea was defined as the occurrence of three or more loose, liquid, or watery stools in a 24-h period. For every recruited case of diarrhea, three control stool samples were collected from children living in the same community, and who had not had diarrhea in the preceding two weeks. The control was age-matched within 2 months for children < 1 year of age and six months for older children.

### Specimen collection and processing

Stool samples were collected and placed in Cary-Blair transport medium, and transported in iced boxes within 4 h to the laboratory at the Universidade Federal do Espírito Santo. Samples were inoculated onto the surface of MacConkey and Hektoen agars for the selection of *E. coli, Shigella,* and *Salmonella* isolates. After incubation for 24 h at 37°C, four lactose-fermenting colonies with typical *E. coli* morphology, and two non-lactose-fermenting colonies were subjected to biochemical tests for identification. In addition, samples were analyzed for the presence of *Giardia lamblia*, *Entamoeba hystolytica*, and *Cryptosporidium*, using standard methods [10]. All *E. coli* strains were maintained in nutrient agar slants at room temperature.

### Multiplex PCR assays

All *E. coli* isolates were subjected to two multiplex PCRs, as previously described, with some modifications [5]. PCR1 assay contained a primer mix for the detection of the following virulence markers: *E. coli* attaching and effacing (*eae*) gene (for detection of typical and atypical EPEC), EAF plasmid (for detection of typical EPEC strains), and the antiaggregation protein transporter gene (*aat*; previously referred to as CVD432 or the AA probe) (for detection of EAEC strains). Primers specific for *afa/daa* for the detection of DAEC strains were subsequently included into this multiplex PCR. PCR2 assay contained primers specific for *elt* and *est* (enterotoxins of ETEC), *ipaH* (invasion plasmid antigen H found in EIEC and *Shigella*), and *stx1* and *stx2* (Shiga toxins 1, 2 and variants of STEC). PCR1 assay identified EAEC, DAEC, and tEPEC by the presence of *eae* and *bfpA*, and aEPEC by the presence of only *eae*. PCR2 assay identified ETEC, EIEC, and STEC.

Three to six bacterial colonies from each stool sample were pooled for template DNA preparation immediately prior to PCR testing, suspended in 300 μL of sterile water, and boiled for 10 min. A 5-μL aliquot of this suspension was added to 50 μL of the PCR mixture (50 mM KCl, 10 mM Tris–HCl [pH 8.3], 1.5 mM MgCl_2_, 2 mM of each deoxynucleoside triphosphate), 1.5 U of AccuPrime *Taq* DNA polymerase, and 5 μM of each set of primers except for the *ipaH* primers, which used 10 μM. The reactions were run in a thermal cycler (model system 2400; Perkin-Elmer Corporation, Norwalk, Conn.) with the following cycling conditions: 94°C for 5 min, 40 cycles of denaturation at 95°C for 1 min, annealing at 58°C (assay 1) or 50°C (assay 2) for 1 min and primer extension at 72°C for 2 min followed by a final extension at 72°C for 7 min. PCR products (10 μL) were visualized after electrophoresis in 2% agarose gels in Tris-borate-EDTA buffer and ethidium bromide staining. In all assays, a mixture of DNA from the prototype EPEC E2348/69, EAEC 042, DAEC C1845, ETEC H10407, EIEC EDL1284, and STEC EDL931 strains served as the positive control, while *E. coli* K-12 DH5α was the negative control [4].

### HEp-2 adherence assay

*E. coli* isolates were subjected to HEp-2 adherence tests by the method originally described by Scaletsky et al. [11], with slight modifications. Briefly, monolayers of 10^5^ HEp-2 cells were grown in Dulbecco modified Eagle medium containing 10% fetal bovine in 24-well tissue culture plates (Falcon Becton Dickinson). Bacterial strains were grown statically in 2 ml of brain heart infusion for 16–18 h. The monolayers were infected with ~3 X 10^7^ bacteria (20 μl of bacterial cultures added to 1 ml of DMEM) and incubated at 37°C for 3 h. The infected monolayers were washed with sterile PBS, fixed with methanol, stained with Giemsa stain, and examined for localized adherence (LA), diffuse adherence (DA), and aggregative adherence (AA).

### DNA hybridization

*E. coli* isolates were tested by colony hybridization with the following DNA probes: EPEC adherence factor EAF (1-kb *BamH*I-*Sal*I fragment of pMAR2 [12], *E. coli* attaching and effacing gene encoding intimin (*eae)* (1-kb *Kpn*I-*Sal*I fragment of pCVD434 [13], CVD432 (the nucleotide sequence of the *Eco*RI-*Pst*I fragment of pCVD432 of EAEC) [14], and *daaC* (associated with the biogenesis of DAEC F1845 adhesin) (390-bp *Pst*I fragment of pSLM852 [15]. DNA probes were prepared from recombinant plasmids containing the DNA probe fragments as inserts. Plasmids were extracted by the method of Birnboim and Doly [16] and digested with appropriate restriction endonucleases, and the appropriate restriction fragments were purified by gel extraction. The DNA fragments were labeled by a random primer extension kit (Rediprime DNA labeling system; Amersham) with [α-^32^P]dCTP. Colony blots were prepared with Whatman 541 filter papers which were then processed and hybridized under string conditions as described previously [17].

### Statistical analysis

The statistical analyses were performed using the SPSS version 17.0 (SPSS Inc., Chicago, IL). A sample size of 100 cases and 300 controls was estimated to have 80% power to detect the symptoms associated with infection, assuming a prevalence of 15% in controls and a 5% significance level. Statistical differences were evaluated by chi-square or Fisher’s exact tests. A p value < 0.05 was considered statistically significant.

## Results

Between August 2007 and September 2008, 560 stool samples were collected from 141 children with diarrhea and 419 healthy controls who resided in 23 communities. Among the population studied, 150 (26.8%) children were aged less than 2 years; 216 (38.6%) and 194 (34.6%) were aged 2−5 years and 6−10 years, respectively. The overall sex distribution was 296 (52.8%) male and 264 (47.1%) female.

*E. coli* isolates (n = 1,943) were categorized into different pathotypes based on the results of two multiplex PCRs (Table 1). Diarrheagenic *E. coli* strains were isolated from 253 (45.2%) children, and were associated with diarrhea in children aged < 5 years (p < 0.001). EAEC (20.9%), DAEC (11.6%), aEPEC (8.7%) were the most frequent pathotypes, followed by LT-ETEC (2.3%), tEPEC (0.5%), EIEC (0.5%), LT/ST ETEC (0.4%), and STEC (0.2%). Other enteric pathogens isolated were *Shigella* (0.7%), *Salmonella* (0.2%), *Giardia lamblia* (4.1%), and *Entamoeba hystolytica* (0.9%). Mixed infections were presented in 22 (15.6%) cases and 12 (2.9%) controls (*P* < 0.05).

**Table 1:** Isolation of pathogens (DEC strains by multiplex PCR) from the stools of children with diarrhea (cases) and children without diarrhea (controls)

**Pathogen**	**Children aged <2 years**		**Children aged 2−5 years**		**Children aged 6−10 years**		
	**Cases**	**Controls**	**Cases**	**Controls**	**Cases**	**Controls**	**Total no. of children**
	**(*n *= 40)**	**(*n *= 110)**	**(*n *= 53)**	**(*n *= 163)**	**(*n *= 48)**	**(*n *= 146)**	**(*n *= 560)**
Diarrheagenic *E. coli*							
aEPEC	7 (17.5)	13 (11.9)	5 (9.4)	13 (7.9)	4 (8.3)	7 (4.8)	49 (8.7)
tEPEC	1 (2.5)	0	0	0	2 (4.2)	0	3 (0.5)
EAEC	20 (50)	25 (22.7)	12 (22.6)	33 (20.2)	4 (8.3)	23 (15.7)	117 (20.9)
DAEC	5 (12.5)	10 (9.1)	15 (28.3)	18 (11.0)^a^	6 (12.5)	11 (7.5)	65 (11.6)
LT-ETEC	2 (5.0)	1 (0.9)	3 (5.7)	2 (1.2)	1 (2.1)	4 (2.7)	13 (2.3)
LT/ST-ETEC	0	0	1 (1.9)	0	1 (2.1)	0	2 (0.4)
EIEC	0	0	2 (3.8)	0	0	1 (0.7)	3 (0.5)
STEC	0	0	0	1 (0.6)	0	0	1 (0.2)
All	35 (87.5)	49 (44.5)^b^	38 (71.7)	67 (41.1)^c^	18 (37.5)	46 (31.5)	253 (45.2)
*Shigella* spp.	1 (2.5)	0	2 (1.4)	1 (0.6)	0	0	4 (0.7)
*Salmonella* spp.	0	1 (0.9)	0	0	0	1 (0.7)	1 (0.2)
*Giardia lamblia*	6 (15)	5 (4.5)	3 (5.7)	8 (4.9)	1 (2.1)	0	23 (4.1)
*Entamoeba histolytica*	0	0	1 (1.9)	3 (1.8)	1 (2.1)	0	5 (0.9)

^a^p = 0.0041; ^b^p = 0.0001; ^c^p = 0.0001, for the comparison between diarrhea and control samples.

*E. coli* isolates were further characterized into EPEC, EAEC, and DAEC pathotypes by HEp-2 adherence pattern and DNA probes. The results were compared with the PCR1 assay (Table 2). Of 145 isolates which yielded the AA pattern, 117 were detected by PCR1, and 99 of these strains reacted with the CVD432 probe; 18 EAEC isolates gave a positive-PCR but probe negative result. Similarly, PCR1 detected 65 of 104 isolates that yielded the DA pattern, and 57 of these strains reacted with the *daaC* probe; only 8 isolates gave a positive-PCR but probe negative result.

**Table 2:** **Detection of enteroadherent *E. coli *(ECC) isolated from cases and controls by genotypic and phenotypic tests**

**Diagnostic test**	**N. (%) of isolates**			
**(*n *=560)**	**EEC**	**EPEC**	**EAEC**	**DAEC**
PCR assay	234 (41.8)	52 (9.3)	117 (20.9)	65 (11.6)
DNA probe	206 (36.8)	50 (8.9)	99 (17.7)	57 (10.2)
HEp-2 adhesion	252 (45)	3 (0.5)	145 (25.9)	104 (18.6)

The combined incidence of EPEC, EAEC, and DAEC (collectively termed enteroadherent *E. coli)* isolates from diarrhea cases and controls is shown in Table 3. Enteroadherent *E. coli* strains were significantly associated with diarrhea, whether detected by phenotypic HEp-2 cell assay or by genotypic assays (p < 0.05). Depending of the assay used, enteroadherent *E. coli* strains were isolated in 45% to 56% of cases, a significantly higher incidence that in controls (p < 0.05). Individually, only DAEC strains, detected either by adherence pattern or by PCR1 or by DNA probe, were significantly associated with diarrhea (p < 0.05), particularly in children aged 2–5 years.

**Table 3:** **Prevalence of enteroadherent *E. coli *(ECC) isolated from cases and controls detected by genotypic and phenotypic diagnostic tests**

**Pathotype**	**Diagnostic test**	**Cases**	**Controls**	**p value**
		**(*n *= 141)**	**(*n *= 419**	
ECC	PCR	79 (56)	155 (36.9)	0.0001
	DNA probe	64 (45.4)	142 (33.9)	0.0156
	HEp-2 adhesion	78 (55.3)	174 (41.5)	0.0046
EAEC	PCR	36 (25.5)	81 (19.3)	0.1210
	DNA probe	27 (19.1)	72 (17.2)	0.6107
	HEp-2 adhesion	40 (28.4)	105 (25)	0.4385
DAEC	PCR	26 (18.4)	39 (9.3)	0.0057
	DNA probe	21 (14.9)	36 (8.6)	0.0369
	HEp-2 adhesion	35 (24.8)	69 (16.5)	0.0336
EPEC	PCR (*eae*)	16 (11.3)	33 (8.3)	0.2280
	PCR (*bfpA*)	1 (0.7)	2 (0.5)	1.0000
	*eae* probe	15 (10.6)	32 (7.6)	0.2923
	EAF probe	1 (10.6)	2 (0.5)	1.0000
	HEp-2 adhesion	1 (0.7)	2 (0.5)	1.0000

## Discussion

Diarrheal disease remains an important public health problem for children in developing areas of Brazil. The importance of *E. coli* as a cause of diarrhea and its attributable fraction to the diarrhea prevalence in these areas was unknown. The present study was performed to determine the prevalence of different diarrheagenic *Escherichia coli* categories in symptomatic and asymptomatic children living in low socioeconomic rural communities in southeastern Brazil.

We employed a two-reaction multiplex PCR assay for the detection of the six *E. coli* pathotypes. The PCR 1 contained primers for amplification of *eae*, EAF, *aat,* and *afa/daa* genes for identification of EPEC, EAEC, and DAEC. The PCR 2 contained primers for amplification of LT, ST, Inv, and Stx genes for detection of ETEC, EIEC, and STEC. We were able to validate the PCR1 with HEp-2 cell adherence assays and specific DNA probes. The different combination of adherence patterns and PCR primers showed that the HEp-2 adherence assay remains the “gold standard” for detection of EAEC and DAEC. Compared with DNA hybridization, our results showed that PCR1 assay could be used instead of the DNA probes as a screening method for “typical” EAEC (*aat*^+^) and “typical” DAEC (*afa/daa*^+^) strains in the clinical laboratory [17].

Our results show a high proportion of EPEC, EAEC, and DAEC (collectively termed enteroadherent *E. coli)* accounting for 92.4% of DEC. EAEC was the most prevalent pathotype in both diarrhea cases and controls, in contrast with several studies showing an association with diarrhea [18,19]. In recent studies conducted in different urban centers of Brazil, EAEC strains were found to be dominant and associated with diarrhea [6-9]. By using the CVD432 probe for the detection of EAEC strains, we found a correlation of 70% sensitivity, which is accordance with the levels of sensitivity found in other studies [6-9]. By using the PCR1, we found a sensitivity and specificity similar to those of the CVD432 probe.

DAEC strains, detected either by adherence pattern or by genotypic assays, were the second most frequently isolated pathotype (11.6-18.6%) and were significantly associated with diarrhea, especially in children aged 2–5 years. Similarly, in a prospective cohort study in a low socioeconomic level peri-urban community in Santiago, Chile [20], DAEC was found in a large proportion of diarrhea cases. In Mayan children in Mexico [21] and aboriginal children in Australia [22], DAEC strains were also predominant and associated with diarrhea. Taken together, these findings suggest that DAEC may be an important enteric pathogen in children living in endemic areas. Our results also support the evidence from studies showing an association of DAEC with age-dependent diarrhea [20,22].

In this study, aEPEC was more common than tEPEC, with is concordance with current data suggesting that aEPEC is more prevalent than tEPEC in both developed and developing countries [4,23]. In Brazil, aEPEC has been increasingly reported and was recently implicated as a cause of diarrhea in different urban centers of Brazil [6-9]. Although differences in the population studied may exist, the prevalence of aEPEC in this and other studies underscores the emergence of aEPEC strains in Brazil.

The prevalence of ETEC was low, in agreement with other studies performed in different parts of Brazil [6-9]. EIEC and STEC were infrequently isolated, suggesting a less important role in diarrhea in Brazilian children [6-9].

## Conclusion

We conclude that enteroadherent *E. coli* strains are involved in a significant proportion of diarrhea cases among children from low socioeconomic level communities. Apart from DAEC whose pathogenic role in diarrhea is still controversial, EAEC was the predominant *E. coli* pathotype. This should be taken as an alert for public health policies since the EAEC pathotype may harbor other very threatening virulence factors such as Shiga toxins. In addition, this study demonstrates that PCR1 assay is an excellent tool for the identification of EAEC and DAEC.

## Competing interests

The authors declare that they have no competing interests.

## Authors’ contributions

LCS and SSK designed the study. FV were responsible for collection of specimens and clinical information. Laboratory investigations and data analysis were performed by DML, TBS, and MMV. ICSA assisted in the development of the research proposal and preparation of manuscript. All authors read and approved the final manuscript.

## Pre-publication history

The pre-publication history for this paper can be accessed here:

http://www.biomedcentral.com/1471-2334/13/418/prepub
